# Mobile Phone Use and Acceptability for the Delivery of Mental Health Information Among Perinatal Adolescents in Nigeria: Survey Study

**DOI:** 10.2196/20314

**Published:** 2021-01-26

**Authors:** Lola Kola, Dolapo Abiona, Adeyinka Olufolake Adefolarin, Dror Ben-Zeev

**Affiliations:** 1 University of Ibadan Ibadan Nigeria; 2 University of Washington Seattle Seattle, WA United States

**Keywords:** mHealth, perinatal adolescent, perinatal depression, community, low income

## Abstract

**Background:**

There are several barriers that may hamper adolescent mothers’ utilization of available health interventions for perinatal depression. Innovative treatment approaches are needed to increase adolescent mothers’ access to mental health care for improved maternal and child health outcomes. Mobile phones have the potential to serve as important conduits to mental health care in Africa. However, mobile phone use patterns and needs among young mothers in Nigeria are not well documented.

**Objective:**

This study sought to determine the prevalence of mobile phone use among perinatal adolescents and report patterns of use, as well as to assess the openness of young mothers to mobile health (mHealth) mental health interventions.

**Methods:**

We surveyed 260 adolescent mothers (ages 16-19 years) in their perinatal or postnatal periods of pregnancies in 33 primary health care clinics in Ibadan, Oyo State, Nigeria in 2020. Respondents were included if they were pregnant with a gestation age of greater than or equal to 4 weeks, or had babies (which they had birthed) that were younger than 12 months.

**Results:**

The total study sample consisted of 260 adolescent mothers with a mean age of 18.4 (SD 0.88) years. The majority of the respondents (233/260, 89.6%) owned mobile phones (eg, keypad, keypad and internet, smartphones); 22 (8.5%) of the 260 mothers had access to phones that belonged to relatives who lived in the same household, while 5 (1.9%) had access only to public paid phones. Only 23% (54/233) of phone owners (which is 20.5% of the total study population) had smartphones. On average, respondents reported first using mobile phones at 15.5 (SD 2.06) years old. The majority of respondents (222/260, 85.4%) reported using their phones for an average of 45 minutes daily for calls to family members. Facebook was the social media platform that was most often used among respondents who had phones with internet access (122/146 minutes per day, 83.4%). The majority of the sample responded as being “interested” and “very interested” in the use of mobile phones for preventive interventions (250/260, 96.2%) and treatment (243/260, 93.5%) information on mental illness such as depression and “hearing voices.” Half of the respondents (126/233, 50.4%) preferred to receive such information in the form of text messages.

**Conclusions:**

Findings from this study suggest that the vast majority of perinatal adolescents in Nigeria own and use mobile phones and that they are interested in leveraging these devices for prevention, treatment, and informational campaigns focused on mental health. The use of smartphones in this population is relatively low, and health intervention through text messages were favored by the women.

## Introduction

Sub-Saharan Africa has the world’s highest level of adolescent childbearing [1]. In Nigeria, up to 31% of women have had a live birth before the age of 18 [1]. The challenges of pregnancy and childbirth increase the vulnerability of expectant mothers to common perinatal mental health problems such as anxiety and depression [2]. Pregnancy in adolescence occurs at a developmental period of intense psychological and physical change, thereby increasing the risk of mental illness [2].

Depression is the most common mental illness in childbearing women [3]. Perinatal depression (ie, depression occurring in pregnancy and up to one year into the postpartum period) is an important health issue affecting women and the emotional, cognitive, and physical development of their children [3]. Perinatal depression negatively impacts maternal-infant bonding, which is necessary for child brain development and growth [3]. In adolescent mothers, perinatal depression increases the risks associated with additional pregnancies, the use of aggressive parenting techniques, and levels of psychopathology in their children [4,5]. Psychosocial treatments delivered in primary care settings in developing countries have shown promise in combatting perinatal depression [6]. However, there are a host of barriers that may hamper young mothers’ access to these interventions in clinical settings [7]. Innovative treatment approaches are needed to increase adolescent mothers’ access to mental health care for improved maternal and child health outcomes. Mobile phones have the potential to serve as important conduits to mental health care in Africa.

There are reports that mobile phone subscriptions in low-to-middle-income countries exceed 95% of the population [8]. In Africa, mobile phone usage cuts across age groups and classes of different socioeconomic statuses [9]. The scale of usage among poor populations is particularly prominent in the sub-Saharan African region [10]. Mobile health (mHealth) approaches are increasingly being used to support mental health care [11] to overcome access related barriers such as cost of treatment, long waiting times at the clinics, and low human resources in health, even among youth populations [12]. However, to design usable and effective mobile interventions, it is important to understand the characteristics and needs of the interventions’ intended users [13].

Available data on mobile phone ownership in Nigeria according to age groups are limited and do not capture the characteristics of vulnerable mothers in primary care [14,15]. To address this need, we surveyed 260 perinatal adolescent mothers receiving primary care services in clinics in Ibadan, Oyo State, Nigeria. The objectives of the study were to ascertain the 1) proportion of perinatal adolescents in this community that use mobile phones, 2) pattern of use of mobile phones in this population, and 3) openness of adolescent mothers to mHealth-supported mental health interventions. Data from this study will inform the design and development of a targeted mHealth intervention for the management of depression in this typically hard to reach population.

## Methods

This project was approved by the University College Hospital/University of Ibadan ethics review board and carried out in 33 antenatal clinics in the 11 local government areas of Ibadan between the 24th of February and the 23rd of March 2020. Consecutive perinatal adolescent attendees in primary care who were between the ages of 16-19 years were approached to participate in the study. Under the Nigerian constitution, persons under 18 years are minors. Notwithstanding, the Child Rights Act makes the provision that a child who is 16 years old has the right to give consent for scientific investigation without parental consent [16]. Four experienced female research assistants fluent in Yoruba invited young perinatal women in the primary care clinics to participate in the survey. Survey questions were in Yoruba and were administered by our research assistants. At every clinic, the research assistants initially obtained information on the age of respondents from clinic records after which viable candidates were approached in the outpatient areas. All survey interviews were conducted on the different antenatal and immunization days of the 33 clinics. Only respondents who were pregnant (gestation age of >4 weeks) or who had babies younger than 12 months were invited to participate in the study. Of the 266 mothers who were approached, 260 agreed to participate in the survey. At the screening stage, before the consent process, 6 mothers refused to participate because they were too upset about being pregnant at young ages. All 260 willing participants were required to provide written, informed consent to participate in the study. Information obtained as part of the survey included basic demographic details of respondents, pattern of mobile phone use, and willingness to engage in the use of mobile phones for general health and mental health–related programs. Interview surveys were read out to participants because of the possible low literacy rate of respondents.

The interview process took an average of 15 minutes. Respondents were given detergent soap that cost an equivalent of 1$ as an incentive. Data entry, validation, cleaning, and analysis were done using SPSS version 15. Descriptive statistics were used to report findings.

## Results

### Demographic Characteristics of Respondents

The total study sample consisted of 260 adolescent mothers with a mean age of 18.4 (SD 0.88) years. The majority of the young women (141/260, 54.2%) were still pregnant, with a mean gestation age of 25.6 (SD 6.2) weeks, while others (119/260, 45.8%) had babies (children less than a year old) with a mean age of 36.2 (SD 26.6) weeks. The majority (204/260, 78.4%) had completed secondary school (12 years of schooling) education, while 17 of the 260 respondents (6.6%) had only a primary school education (6 years of schooling). Of the 260 respondents, 129 (49.5%) were artisans (tailors and hairdressers) and petty traders. Only 23 (8.8%) were married, whereas 157 (60.4%) were cohabiting and 80 (30.8%) were single.

### Ownership, Usage, and Access to Mobile Phones

The majority of the respondents (233/260, 89.6%) owned mobile phones. The majority (54/233, 23.2%) of the owned phones were smartphones, whereas 92 of the 233 phones (39.5%) were keypad phones with internet functions (mostly with preloaded Facebook and gaming apps), and 87 (37.3%) were basic phones. The choice of mobile network was determined mostly by network coverage in the areas the respondents lived (133/233, 57.1%), and reduced cost of airtime and mobile data (89/233, 38.2%). More than three-quarters of the respondents (195/233, 83.7%) reported their phones as personally owned and not shared with others. On average, respondents reported 15.5 years as the age when they first started using a mobile phone. Out of the 260 total respondents, 27 (10.4%) did not owe a mobile phone, and 22 (8.5%) had access to phones that belonged mostly to relatives who lived in the same house with them for an average of 80 minutes per day. Phone ownership and use demographics are depicted in Table 1.

**Table 1:** Mobile phone ownership, privacy, and access among respondents (N=260).

Variable		Frequency (%)
**Type of phone owned (n=233)**		
	Android	54 (23.2)
	Keypad phones with internet access	92 (39.5)
	Keypad phone	87 (37.3)
**Type of network (n=255, multiple responses per respondent permitted)**		
	Airtel (free internet mode and bonus airtime)	138 (53.1)
	MTN (bonus airtime)	53 (20.4)
	Glo	61 (23.5)
	9mobile/Etisalat	3 (1.2)
**Why choose this** **network (n=233)**		
	Affordable (airtime and mobile data)	89 (38.2)
	Convenient (network coverage in area lived)	133 (57.1)
	No reasons	11 (4.7)
**How often do you top up your mobile phone (n=233)**		
	Weekly	164 (70.3)
	Bi weekly	27 (11.6)
	>Monthly	42 (18.1)
**Do others have access to the information on your phone (n=233)**		
	Yes	38 (16.3)
	No	195 (83.7)
Age when you had your first phone, mean years (SD) (n=233)		15.5 (2.1)
**Do you have access to a mobile phone that is not yours (n=27)**		
	Yes	22 (8.5)
	No	5 (1.9)
**If yes to the above, to whom does the phone belong (n=22)**		
	Relative living in the same house	16 (72.7)
	Friends/neighbours not living in the same house	6 (27.3)
Time you have access to phones that is not yours per day, mean minutes (SD) (n=22)		80 (79)

### Mobile Phone Usage

Table 2 shows the pattern of mobile phone use among respondents. The majority of respondents (222/260, 85.4%) used their phones for a daily average of 45 minutes, mostly to call family members. Texting (SMS) was the least prevalent activity (23/260, 8.8%) at an average of 12 minutes per day. Facebook was the most used social media site among respondents (122 average minutes per day, 83.4%), and only a minority of respondents reported using WhatsApp. Also, only 8 (3.1%) of the 260 respondents reported that they conducted Google searches; however, these respondents reported an average of 110 minutes of daily Google searches. The average times spent by respondents on different activities were 112 minutes using Facebook, 104 minutes using WhatsApp, 110 minutes Google searching, and 112 minutes watching movies online.

**Table 2:** Mobile phone patterns of use among perinatal adolescents (N=233).

Variable		Frequency, minutes/day (%)	Mean (SD)
**Who do you call most**		222 (85.4)	45 (40)
	Husband/boyfriend	104 (46.8)	—^a^
	Family members	100 (45.0)	—
	Friends	13 (5.9)	—
	In laws	5 (2.3)	—
**Who do you text most**		23 (8.8)	12 (11)
	Husband/boyfriend	11 (47.8)	—
	Family members	3 (13.0)	—
	Friends	7 (30.4)	—
	In law	2 (8.7)	—
**Internet use**			
	Facebook	122 (83.6)	112 (92)
	WhatsApp	23 (15.7)	104 (89)
	Instagram	1 (0.7)	10 (10)
	Other ( Google)	8 (3.1)	110 (86)
	Watching movies online	100 (38.5)	122 (80)
**Playing games**		27	75 (46)

^a^Not available.

### Interest in mHealth

When asked if they would like general health information (how to take care of themselves during pregnancy) delivered to them via mobile phone, almost all of the respondents (253/260, 97%), even including those that did not own a mobile phone, responded affirmatively (Table 3). The majority also responded as being “interested” and “very interested” in the use of mobile phones for preventive information (250/260, 96.2%) and treatment information (243/260, 93.5%) on mental illness such as depression and “hearing voices.” Half of the respondents (126/260, 50.4%) preferred to receive such information as text messages, while very few (26/260, 10.4%) preferred such information as videos on phone apps.

**Table 3:** Perinatal adolescents’ interest in mHealth (N=260).

Variables		Frequency, n (%)	
**Would you like for mobile phones to be used to deliver health information to you**			
	Yes	253 (97.3)	
	No	7 (2.7)	
**Would you like it if the mobile phone is used to deliver health information on prevention of mental disorder such as depression or hearing voices to you**			
	Yes	250 (96.2)	
	No	10 (3.8)	
**Would you like it if the mobile phone is used to deliver health information on treatment of mental disorder such as depression or hearing voices to you**			
	Yes	243 (93.5)	
	No	17 (6.5)	
**How would you like such information to be delivered**			
	Phone calls	97 (38.8)	
	Text messaging	126 (50.4)	
	Short videos on an app	26 (10.4)	
	Named social networking platform (WhatsApp and Facebook)	1 (0.4)	

## Discussion

### Principal Findings

To our knowledge, this is the first study that systematically summarizes the use of mobile phones among perinatal adolescents in primary care in Africa and the first to document the potential viability of mHealth use for mental health care delivery in this population. Primary care clinics in Nigeria are first-line public health facilities that serve the country’s grassroots. A significant strength that increases the accuracy of our findings is its representation of community-dwelling, low-income perinatal adolescents from 33 antenatal clinics in Oyo state, southwest Nigeria. The research assistants were experienced in data collection among perinatal women in primary care and had no notable problem inviting and engaging perinatal adolescents to participate in the study.

There are several findings from this study that can inform mHealth innovations in maternal mental health care for young mothers in low-income settings. Our results showed that most adolescent mothers are adequately educated. This finding is contrary to previous findings that associates adolescent pregnancy with low literacy rates [17], limited educational attainment, and limited livelihood opportunities [18]. The level of education of adolescent mothers has implications for the viability of various mHealth interventions in this population. Considering their age group and level of education, text-based resources assisted with audio-visual tools would be a possible way to enhance user engagement of mHealth interventions [19]. Almost all of the young mothers interviewed owned or had access to a mobile phone, which they used every day [14]. Mobile phones owned included inexpensive smartphones that can host apps; keypad phones that can support internet searches, and other basic keypad phones with call, text, and radio functions. A minority of the respondents who did not have phones had access to their relatives’ phones. This has implications for patient privacy in mHealth treatments. In such instances, it may be that young mothers would be required to type in passwords to access treatment information on a mobile phone app. There will also be a need for auto-lock functions immediately after use to prevent secondary users’ access to what might be private health information.

Nine out of ten mobile phone users’ choice of mobile network services was influenced by network coverage in the area they lived. Respondents also considered the availability of network facilities such as airtime credit, unlimited free data, airtime bonus, and night browsing modes [20]. This might also be useful in the choice of mobile networks in the planning of an mHealth intervention to allow incentives for the use of mobile phones. All respondents used a pay-as-you go method (ie, calling cards). More than two-thirds of the mobile phone owners topped up their network credit weekly at an average of approximately three-quarters of a US dollar to allow them to stay in contact with family members and friends.

Different competing data bundle plans by telephone network services in the country [21] allowed more than half of the respondents to use network data on the mobile phone. This is an important consideration when exploring potential mHealth approaches that require internet access for this population. This information suggests that if internet-based functions are required in an intervention model, it might be necessary to make provisions for network data because many of the respondents that use internet access rely on the free data bonus and free night browsing made possible by mobile network providers (a reason for the respondents’ choice of networks). Only 23% (54/233) of phone owners (ie, 20.5% of the sampled population) had smartphones, which would mean smartphone apps for intervention in this population would lead to the exclusion of many adolescent perinatal women with depression. However, smartphones are becoming more widely used in Nigeria [22], which may suggest greater potential for app-based interventions in this population in the future. Further research is needed to enquire about this population’s willingness to engage in mHealth intervention models that are installed on the smartphone for offline use. Facebook was reported as the preferred social media tool among our respondents, a finding that is consistent with research conducted among low-income youth [23]. Also, although few social media sites were visited by adolescent mothers, as similarly reported among disadvantaged youth communities [24], patronage of a wider range of social media sites has been reported among university students in Nigeria [15]. More than half of the survey respondents who were mobile phone owners reported the use of social media on a daily basis for over an hour, a duration that is not dramatically different from previous reports in the country [15]. Nine out of ten respondents endorsed the use of the mobile phone for delivery of health information. When asked specifically about mobile phone use for information on prevention and treatment of mental disorders such as depression or mental disorders that cause auditory hallucinations, nine out of ten respondents were also interested in mHealth information delivery. These findings support a broad willingness to engage in mHealth initiatives for the delivery of care for mental illness among perinatal adolescents. Despite the pattern of mobile phone use among respondents, which indicated making calls as a prevalent activity and online videos as the second favorite internet activity, adolescents had a preference for mHealth messages in the form of text messages, perhaps because text messages would not require the use of their network credit or perhaps due to their desire for flexibility and convenience during mHealth interactions [25].

### Limitations

As in all survey research, our findings are based on respondents’ self-reports. Thus, our study is susceptible to inaccurate recall or unrepresentative reporting. Social desirability bias could have been a major limitation of this research. Data was self-reported, and respondents might have given what they viewed were socially acceptable responses. The majority of the questions were structured, and respondents might have chosen answers that did not totally reflect their views but instead reflected views of the researchers who constructed the survey.

### Conclusions

The study suggests low-income perinatal adolescents are open to engaging in mHealth use for mental health service delivery. A variety of mobile phone-based interventions including those with audio-visuals can be considered as promising in this population because of the population’s limited literacy rate. Smartphone access is currently limited in this population and equity issues will relate to use of the phone-based interventions with limited technology to maximize access. The majority of the young mothers we surveyed had access to mobile technology that would be necessary for the implementation of a successful mobile phone-based intervention.

## References

[1] Nigeria NBOS (2011). Nigeria multiple indicator cluster survey (MICS) - 2011 Monitoring the situation of children and women. Nigeria National Bureau of Statistics.

[2] Kingston D, Heaman M, Fell D, Chalmers B, Maternity Experiences Study Group of the Canadian Perinatal Surveillance System‚ Public Health Agency of Canada (2012). Comparison of adolescent, young adult, and adult women's maternity experiences and practices. Pediatrics.

[3] Satyanarayana Veena A, Lukose Ammu, Srinivasan K (2011). Maternal mental health in pregnancy and child behavior. Indian J Psychiatry.

[4] Elfenbein DS, Felice ME (2003). Adolescent pregnancy. Pediatric Clinics of North America.

[5] Hodgkinson S, Beers L, Southammakosane C, Lewin A (2014). Addressing the mental health needs of pregnant and parenting adolescents. Pediatrics.

[6] Oladeji BD, Bello T, Kola L, Araya R, Zelkowitz P, Gureje O (2019). Exploring Differences Between Adolescents and Adults With Perinatal Depression-Data From the Expanding Care for Perinatal Women With Depression Trial in Nigeria. Front Psychiatry.

[7] Kola L, Bennett Ian M, Bhat Amritha, Ayinde Olatunde O, Oladeji Bibilola D, Abiona Dolapo, Abdumalik Jibril, Faregh Neda, Collins Pamela Y, Gureje Oye (2020). Stigma and utilization of treatment for adolescent perinatal depression in Ibadan Nigeria. BMC Pregnancy Childbirth.

[8] Sustainable DG, Statistics IROMCS Sustainable Development Goals. Mobile-cellular subscriptions​.

[9] Silver L (2019). and C. Johnson, Majorities in sub-Saharan Africa own mobile phones, but smartphone adoption is modest, Pew Research Centre, viewed.

[10] Porter G, Hampshire K, Abane A, Munthali A, Robson E, Bango A, de Lannoy A, Gunguluza N, Tanle A, Owusu S, Milner J (2015). Intergenerational relations and the power of the cell phone: Perspectives on young people’s phone usage in sub-Saharan Africa. Geoforum.

[11] Ben-Zeev D (2014). Technology-based interventions for psychiatric illnesses: improving care, one patient at a time. Epidemiol Psychiatr Sci.

[12] Fedele DA, Cushing CC, Fritz A, Amaro CM, Ortega A (2017). Mobile Health Interventions for Improving Health Outcomes in Youth: A Meta-analysis. JAMA Pediatr.

[13] Gudmundsdottir G (2014). B. and K. B. Vasbø, Methodological challenges when exploring digital learning spaces in education: Springer.

[14] Akinfaderin-Agarau F, Chirtau M, Ekponimo S, Power S (2012). Opportunities and limitations for using new media and mobile phones to expand access to sexual and reproductive health information and services for adolescent girls and young women in six Nigerian states. Afr J Reprod Health.

[15] Neier S, Zayer LT (2015). Students’ Perceptions and Experiences of Social Media in Higher Education. Journal of Marketing Education.

[16] United Nations RON (2010). Committee on Rights of Child examines report of Nigeria. UN Committee on the Rights of the Child.

[17] Fleming N, O’Driscoll T, Becker G, Spitzer RF, Allen L, Millar D, Brain P, Dalziel N, Dubuc E, Hakim J, Murphy D, Spitzer R (2015). Adolescent Pregnancy Guidelines. Journal of Obstetrics and Gynaecology Canada.

[18] Chiazor A (2017). Teenage pregnancy: The female adolescent dilemma. International Journal of Science Commerce and Humanities.

[19] Ben-Zeev D, Brian RM, Aschbrenner KA, Jonathan G, Steingard S (2018). Video-based mobile health interventions for people with schizophrenia: Bringing the “pocket therapist” to life. Psychiatric Rehabilitation Journal.

[20] Omotayo F (2018). O. and O. V. Abolade, Determinants of subscribers? choice of internet service providers in Nigeria. JORIND December. ISSN.

[21] Cent (2015). , P. R., Center PR. Cell phones in Africa: Communication lifeline.

[22] Pew C (2017). Smartphone ownership and Internet usage continues to climb in emerging economies. emerging-economies/ accessed 1 November.

[23] Micheli M (2016). Social networking sites and low-income teenagers: between opportunity and inequality. Information, Communication & Society.

[24] Stevens R, Gilliard-Matthews S, Dunaev J, Woods MK, Brawner BM (2017). The Digital Hood: Social Media Use among Youth in Disadvantaged Neighborhoods. New Media Soc.

[25] Committee on Adolescence American Academy of Pediatrics (2008). Achieving quality health services for adolescents. Pediatrics.

